# X-ray crystallography and sickle cell disease drug discovery—a tribute to Donald Abraham

**DOI:** 10.3389/fmolb.2023.1136970

**Published:** 2023-05-24

**Authors:** Akua K. Donkor, Piyusha P. Pagare, Mohammed H. AL Mughram, Martin K. Safo

**Affiliations:** Department of Medicinal Chemistry and the Institute for Structural Biology, Drug Discovery and Development, School of Pharmacy, Virginia Commonwealth University, Richmond, VA, United States

**Keywords:** X-ray crystallography, structure-based drug discovery, aromatic aldehydes, sickle cell disease, hemoglobin, oxygen affinity, antisickling, allosteric effectors

## Abstract

X-ray crystallography and structure-based drug discovery have played a major role in the discovery of antisickling agents that target hemoglobin (Hb) for the treatment of sickle cell disease (SCD). Sickle cell disease, the most common inherited hematologic disorder, occurs as a result of a single point mutation of βGlu6 in normal human adult hemoglobin (HbA) to βVal6 in sickle hemoglobin (HbS). The disease is characterized by polymerization of HbS and sickling of red blood cells (RBCs), leading to several secondary pathophysiologies, including but not limited to vaso-occlusion, hemolytic anemia, oxidative stress, inflammation, stroke, pain crisis, and organ damage. Despite the fact that SCD was the first disease to have its molecular basis established, the development of therapies was for a very long time a challenge and took several decades to find therapeutic agents. The determination of the crystal structure of Hb by Max Perutz in the early 60s, and the pioneering X-ray crystallography research by Donald J. Abraham in the early 80s, which resulted in the first structures of Hb in complex with small molecule allosteric effectors of Hb, gave much hope that structure-based drug discovery (SBDD) could be used to accelerate development of antisickling drugs that target the primary pathophysiology of hypoxia-induced HbS polymerization to treat SCD. This article, which is dedicated to Donald J. Abraham, briefly reviews structural biology, X-ray crystallography and structure-based drug discovery from the perspective of Hb. The review also presents the impact of X-ray crystallography in SCD drug development using Hb as a target, emphasizing the major and important contributions by Don Abraham in this field.

## 1 Introduction

### 1.1 Structural biology and structure-based drug discovery

Structural biology has become an indispensable tool for determining the three-dimensional (3D) structures of macromolecules, e.g., proteins for a comprehensive understanding of their functions on molecular level, as well as a detailed atomic level description of potential binding cavities that can be targeted for structure-based drug design or discovery (SBDD). Traditionally, X-ray crystallography and to a lesser extent nuclear magnetic resonance (NMR) have been the two major structural biology techniques used to study atomic structures, providing us with a tremendous number of macromolecule 3D structures. The field of structural biology and structure-based drug discovery can be attributed to Max Perutz and John Kendrew for using X-ray crystallography to solve the structures of hemoglobin and myoglobin, respectively, at atomic resolutions (Kendrew et al., 1958; Perutz et al., 1960). These incredible feats won the two scientists the Nobel Prize in Chemistry in 1962. While X-ray crystallography yields the highest atomic resolution of protein structures, it has several drawbacks, including the need for a pure, stable, and crystallizable protein in sufficient amounts. Larger proteins and/or membrane proteins are much more difficult to express and crystallize, making target proteins larger than 250 kDa challenging to solve by X-ray crystallography (Davis et al., 2003). Another major limitation of X-ray crystallography is its inability to properly model protein dynamics; rather capturing a static state that may or may not be the relevant physiological state of interest. Unlike X-ray crystallography, NMR does not require the protein to be crystallized, and most importantly captures the structure of proteins in solution; providing unique information about dynamics and multiple states. NMR also has an advantage over X-ray crystallography as it can be used to obtain high resolution structural information from unstructured proteins. NMR, however, requires highly pure proteins and is usually unsuitable for large macromolecules, although relatively large structures have been solved using this technique. Nonetheless, for small proteins NMR could be faster and practical than X-ray crystallography. Another bottleneck of NMR is the requirement of isotopic labeled proteins (e.g., ^13^C and ^15^N-labelled proteins) for easier and reliable analysis. It is notable to point out that several Nobel Prizes directly related to NMR spectroscopy have been awarded (Boesch, 2004). In 1991, the Nobel Prize in chemistry was awarded to Richard Robert Ernst for his contribution to the development of the methodology of high-resolution NMR spectroscopy. In 2002, Kurt Wüthrich was also awarded the Nobel Prize in chemistry for his development of NMR spectroscopy for determining the 3D structure of biological macromolecules in solution. Felix Bloch and Edward Mills Purcell shared the Nobel Prize award in Physics in 1952 for their development of new methods for nuclear magnetic precision measurements. The limitations of X-ray crystallography and NMR methodologies have raised demand for another structural biology technique, i.e., cryo-electron microscopy (Cryo-EM), which has shown remarkable growth in the last decade or so due to continuing improvements in hardware and software (Vénien-Bryan et al., 2017; Renaud et al., 2018; Drie and Tong, 2020; de Oliveira et al., 2021). Currently, Cryo-EM can routinely produce structures with high atomic resolutions as X-ray crystallography and considering that Cryo-EM structures are determined close to their native state, gives much hope for its application in structure-based drug design. Furthermore, the method has improved tremendously allowing for imaging or visualizing macromolecular complexes in their functional cellular context (Cheng, 2018; Drie and Tong, 2020; de Oliveira et al., 2021). The scientific world took notice of Cryo-EM technique and awarded the 2017 Nobel Prize in Physics to Dr. Jacques Dubochet, Dr. Richard Henderson, and Dr. Joachim Frank (Elbaum et al., 2021).

Currently (as of 1 March, 2023), there are over 203,000 macromolecular structures deposited in the Protein Data Bank (PDB), with about 174,000 determined using X-ray crystallography, about 14,000 each with NMR and Cryo-EM (http://www.pdb.org/pdb/search/advSearch.do). In particular, X-ray crystallography has played a pivotal role in understanding Hb allostery and for sickle cell disease drug discovery, with Don Abraham as one of the pioneer in the use of this technique (Abraham et al., 1982; Abraham et al., 1983a; Abraham et al., 1983b; Abraham et al., 1984a; Abraham et al., 1984b; Abraham, 1984). NMR and Cryo-EM techniques have only found very little use in Hb structural studies, although the former played a significant role in recognizing the co-existence of multi-liganded relaxed Hb states in equilibrium that include the classical R-state (Lukin et al., 2003; Gong et al., 2006). About 3,600 hemoglobin structures have been deposited in the PDB, the large majority solved using X-ray crystallography (http://www.pdb.org/pdb/search/advSearch.do).

The 3D structures of macromolecules cannot always be experimentally determined, necessitating the use of computational methods, e.g., homology modeling, threading, and *ab initio* techniques to “*predict*” the structures. In recent years, macromolecule structures predicted by computational methods have gained a lot of attention, with the advent of deep-learning approaches (Lavecchia, 2019; Patel et al., 2020; Gupta et al., 2021). The EMBL-EBI (https://alphafold.ebi.ac.uk/) reports over 200,000,000 structures predicted using the deep-learning computational method, AlphaFold, an Artificial Intelligence (AI) system developed by DeepMind to predict 3D structures of macromolecules from their amino acid sequences (Jumper et al., 2021). This technique is a vast improvement over existing macromolecule computational techniques because of its ability to predict structures with atomic accuracy even in the absence of a known similar structure (Jumper et al., 2021). It begun in 2018, when DeepMind introduced AlphaFold at the Critical Assessment of Protein Structure Prediction (CASP13) competition, where it achieved remarkable success in predicting the 3D structures of proteins, outperforming all other methods. AlphaFold was later updated and re-released as AlphaFold2 in 2020, which further improved the accuracy of protein structure prediction (Jumper et al., 2021). The development of AlphaFold represents a significant breakthrough in the field of structural biology, offering a powerful new tool for protein structure prediction and advancing our understanding of biological systems with implications for drug discovery, disease research, and basic biological understanding.

Structure-based drug design remains one of the most powerful and logical approaches in drug discovery paradigms, allowing for targeted, efficient, and rapid process for lead discovery and optimization. Paul Ehrlich, who won a Nobel prize in Chemistry in 1908 is credited for envisioning receptors as atomic locks and drugs as atomic keys (Piro et al., 2008). Structure-based drug design revolves around structural biology with X-ray crystallography, historically and presently, playing the most significant role. A key step in SBDD is determination of the crystal structure of the macromolecule target in complex with a ligand, e.g., endogenous substrate or product, drug or effector or inhibitor or substrate analog, or a library of hit or fragment molecules. Atomic level information from the complex structure allows for targeted structural modification(s) of the bound ligand that may improve the pharmacologic activity of the ligand. This process can be iterative until a more active agent is discovered. Not only rational design for activity, but other moieties could be incorporated that may regulate the selectivity, solubility, metabolism, and toxicity profile of the lead compound. Beside structural biology, SBDD requires integration of a number of other independent techniques, including but not limited to computational or molecular modeling, chemical biology, synthetic organic chemistry, medicinal chemistry, molecular biology, pharmacology and pharmacokinetics/ADME.

### 1.2 Hemoglobin—the protein of life

Hemoglobin is a multifunctional allosteric protein found in red blood cells (RBC), primarily involved in oxygen transport (Helms and Kim-Shapiro, 2013; Ahmed et al., 2020). It has a molecular weight of about 64,500 Da and is formed by pairing of a heterodimer of the polypeptide chains, α- and β-globins, into a tetrameric functional unit α2β2 (α_1_β_1_-α_2_β_2_). The MetHb or H_2_O-liganded Hb from horse was the first Hb structure to be solved, (Perutz et al., 1960; Perutz et al., 1968; Ladner et al., 1977), followed later by that of unliganded Hb or deoxygenated Hb (DeoxyHb) from human (Muirhead and Perutz, 1963; Bolton and Perutz, 1970; Muirhead and Greer, 1970). The two αβ-dimers are arranged around a two-fold axis of symmetry, resulting in a central water cavity. There are two openings to the central water cavity; an α-cleft (formed between the opposite α-subunits) and a β-cleft (formed between the two β-subunits) (Richards and Wyckoff, 1973; Schechter, 2008; Safo et al., 2011). All four subunits contain Fe-bound heme groups that bind and transport ligands, e.g., oxygen. Although both liganded and unliganded Hb are tetramers, they are characterized by different quaternary structures, resulting in several different unique structural features, most notably different central water cavity size (with that of the unliganded structure larger than the liganded structure), different heme positions, different inter-subunit hydrogen-bond/salt bridge interactions, and different α_1_β_2_ dimer interface (Perutz, 1976; Perutz et al., 1998; Safo et al., 2011). DeoxyHb is referred to as the T-state due to the numerous salt-bridge/hydrogen-bond interactions that stabilize the structure in the tense (T) conformation, while liganded Hb, which is characterized by the absence of most of the T-state salt-bridge/hydrogen-bond interactions is referred to as the relaxed or R-state (Perutz, 1976; Perutz et al., 1998; Safo et al., 2011; Ahmed et al., 2020).

**FIGURE 1:**
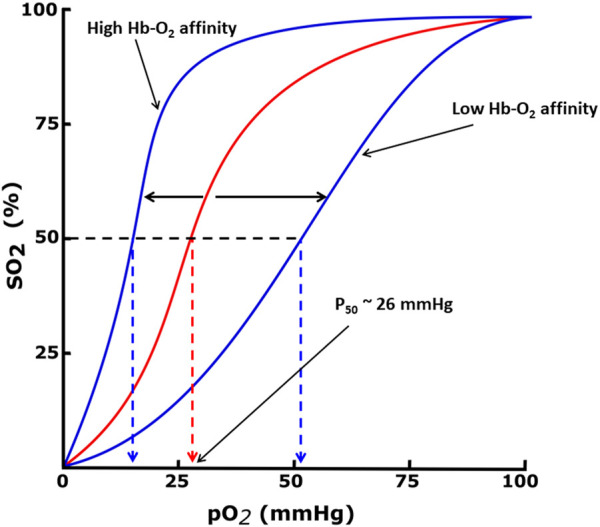
Oxygen equilibrium curve (OEC) of Hb. The normal P_50_ value (∼26 mmHg) is indicated by red dashed lines. The left shift and right shift in the curves (blue) are associated with various conditions, including allosteric effectors of Hb.

**FIGURE 2:**
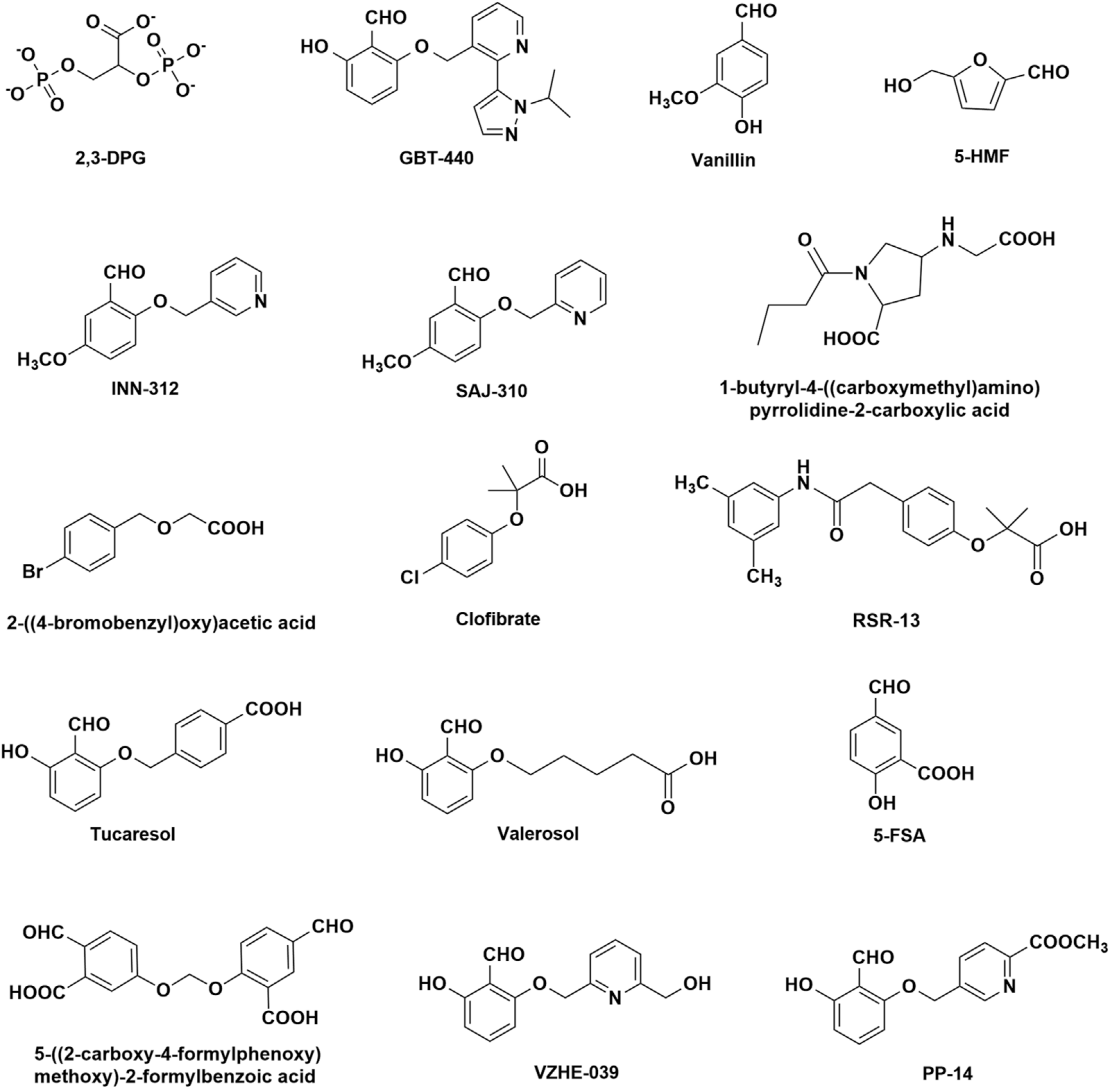
Chemical structures of natural and synthetic allosteric effectors of Hb.

Hemoglobin function is made possible because of its allosteric nature, equilibrating between the unliganded or deoxygenated form (T-state) possessing low oxygen affinity, and the liganded or oxygenated form (R-state), possessing high oxygen affinity (Perutz, 1972; Perutz et al., 1993; Schechter, 2008; Pittman, 2011; Safo et al., 2011; Ahmed et al., 2020). The oxygen affinity of Hb is reported as P_50_, the partial pressure of oxygen (pO_2_) in mmHg at which 50% Hb is saturated with oxygen (sO_2_), and n is the Hill’s coefficient that measures the cooperativity of oxygen binding (Berg et al., 2002; Mozzarelli and Bettati, 2011; Safo et al., 2011). The P_50_ value of normal hemoglobin is approximately 26 mmHg. The quantitative relationship between the pO_2_ and sO_2_ can be represented by the oxygen equilibrium curve (OEC) or the oxygen dissociation curve (ODC) (Figure 1) (Berg et al., 2002; Schechter, 2008; Pittman, 2011) A right-shift of the OEC or decrease in Hb affinity for oxygen (low Hb-O_2_ affinity) leads to an increase in P_50_, while the converse is true. Hb oxygen transport function is aided by the endogenous allosteric effector, 2,3-diphosphoglyceric acid (2,3-DPG; Figure 2), which right-shifts the OEC and allows for effective delivery of oxygen from the protein to cells to facilitate aerobic respiration and energy production (Figure 1) (Schechter, 2008; Pittman, 2011; Safo et al., 2011; Ahmed et al., 2020; Safo, 2021; Pagare et al., 2022) 2,3-DPG is able to decrease the oxygen affinity of Hb by preferentially binding to the β-cleft of DeoxyHb, several angstroms from the ligand binding heme pocket. Binding of 2,3-DPG ties the two β-subunits together through hydrogen-bond/salt bridge interactions that stabilize the T-state Hb relative to the R-state Hb (Benesch and Benesch, 1969; Bunn and Briehl, 1970; Bunn and Jandl, 1970; Arnone, 1972; Macdonald, 1977; Richard et al., 1993).

**FIGURE 3:**
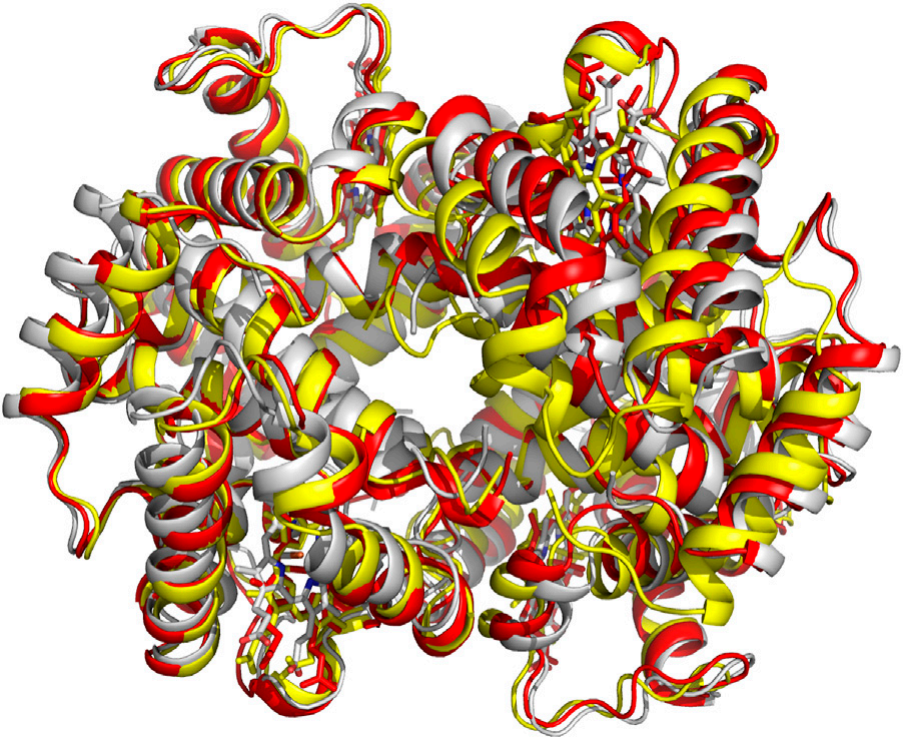
Crystal structures of classical R-state (red), R2-state (gray) and R3-state (yellow) superposed on each other. Hb subunits and hemes are in ribbons and sticks, respectively.

The crystal structures of both the liganded and deoxygenated Hb, in the classical T and R-state conformations, were used to formulate the two-state Monod-Wyman-Changeux (MWC) model, which assumes that upon ligand binding the T-state allosterically switches to the R-state without intermediate states (Monod et al., 1965). An alternative proposed Koshland-Némethy-Filmer (KNF) model assumes that in the absence of ligand, Hb exists in only one conformation and ligand binding induces a conformational change that are transmitted to other subunits (Koshland et al., 1966). Following these two classical models, Perutz proposed a stereochemical model embodying aspects of both the MWC/KNF models to explain the binding of oxygen to Hb, as well as its cooperativity effect (Perutz et al., 1998). Over the years, several modifications and/or variations of allosteric models have been proposed to address apparent shortcomings in the earlier models. Some examples are the Cooperon model of Brunori et al. (1986), the SK model of Szabo and Karplus (1972), the tertiary two-state (TTS) model of Henry et al. (2002), and the global allostery model by Yonetani et al. (2002). This topic has been reviewed extensively, and the reader is referred to one such published article by Eaton et al. (2007). A scientific achievement by Abraham and his colleagues is using X-ray crystallography to show that the R-state Hb is actually an ensemble of relaxed states, e.g., R2, RR2, RR3 and R3, each with an unique quaternary structure (Figure 3), (Mueser et al., 2000; Safo and Abraham, 2001; Safo et al., 2002b; Safo and Abraham, 2003; Safo et al., 2005; Jenkins et al., 2009; Safo et al., 2011) which they took advantage of to design and develop molecules to treat cardiovascular diseases, including sickle cell disease (Safo et al., 2011; Ahmed et al., 2020; Safo et al., 2021; Pagare et al., 2022).

### 1.3 X-ray crystallography in hemoglobin research

X-ray crystallography is the most well-established tool in structural biology and continues to be the primary means to characterize 3D structures of proteins and other macromolecules, (Drenth, 2007) although as pointed out above Cryo-EM technique is increasingly becoming popular. In particular, and as relates to Hb, X-ray crystallography has been fundamental in the formulation of basic theories concerning the behavior of allosteric proteins, understanding the allostery of Hb, (Monod et al., 1965; (Koshland et al., 1966; Szabo and Karplus, 1972; Perutz, 1976; Brunori et al., 1986; Perutz et al., 1998; Mueser et al., 2000; Safo and Abraham, 2001; Safo et al., 2002b; Henry et al., 2002; Yonetani et al., 2002; Safo and Abraham, 2003; Safo et al., 2005; Eaton et al., 2007; Jenkins et al., 2009; Safo et al., 2011; Ahmed et al., 2020; Safo et al., 2021) and in recent years for structure-based drug discovery to treat a number of diseases, including sickle cell disease (Safo et al., 2011; Safo and Kato, 2014; Oder et al., 2016; Ahmed et al., 2020; Safo et al., 2021).

The ability to produce enough and pure protein, either from a biological source or through recombinant means, as well as able to crystallize the protein is a prerequisite for successful crystal structure determination using X-ray crystallography. Fortunately, normal Hb or variant Hb for X-ray crystallography is usually obtained from the biological source, blood (Safo and Abraham, 2001). Hb is one of the most abundant protein in human, forming about 35% and 95% total content and dry weight content of RBC, respectively, thus making it easier to purify a large amount, (Weed et al., 1963; Kaza et al., 2021), especially normal Hb using ion-exchange chromatography (Safo and Abraham, 2001; Beatriz de la Calle Guntiñas et al., 2003). Most variants, on the other hand occur in significantly lower quantity of the total RBC content, requiring extra effort to get adequate amount of pure protein for structural studies. Hb produced by recombinant means, especially Hb variants, have also been used for X-ray crystallography (Hoffman et al., 1990; Hui et al., 1999; Vásquez et al., 1999; Kavanaugh et al., 2001; Cheng et al., 2002; Inayat et al., 2006; Villarreal et al., 2008; Ahmed et al., 2020).

Many factors influence protein crystallization process, e.g., pH, temperature, protein and precipitant concentration, as well as protein purity and stability (Parker, 2003). For most Hb, the concentration for obtaining diffraction quality crystals ranges between 20 and 60 mg/mL, with liganded Hb crystals normally requiring 20–40 mg/mL protein concentration, while unliganded Hb (deoxygenated Hb or DeoxyHb) or T-state crystals may require 40–60 mg/mL. (Safo and Abraham, 200) DeoxyHb is usually crystallized from high-salt (>2.8 M sulfate/phosphate) or low-salt (e.g., polyethylene glycol) precipitants (Safo and Abraham, 2001). As noted above, liganded Hb is characterized by a multi-relaxed states that are conformationally distinct, including the classical R-state (first liganded Hb structure to be solved), as well as R2-state, R3-state, RR2-state, or RR3-state (Abraham et al., 1995; Mueser et al., 2000; Safo et al., 2002b; Safo and Abraham, 2003; Safo et al., 2005; Jenkins et al., 2009; Ahmed et al., 2020). The R3-state, RR2-state, or RR3-state generally crystallizes under high-salt conditions, while the R2-state crystallizes under both low- and high-salt conditions (Mueser et al., 2000; Safo et al., 2002b; 2005; Jenkins et al., 2009; Ghatge et al., 2016; Ahmed et al., 2020). This is an example of how X-ray crystallography had been used to identify several static but snapshots of conformationally different allosteric states of a protein. It is also notable that NMR spectroscopy at near-physiological conditions had previously predicted that liganded Hb exists as a mixture of relaxed states, consistent with the crystallographic data (Lukin et al., 2003; Gong et al., 2006). Unless noted otherwise, henceforth the term R-state is used to denote the ensemble of relaxed states.

The most common approach to crystallizing Hb is using the batch method; made possible because of the availability of large amount of protein (Perutz et al., 1968; Safo and Abraham, 2001). DeoxyHb crystallization experiment must be conducted under inert atmosphere, usually in a glove box, with oxygen free buffers and precipitants to prevent oxidation to H_2_O-ligated ferric Hb or methemoglobin (MetHb) (Perutz et al., 1968; Safo and Abraham, 2001). Crystallization of liganded Hb is usually conducted with carbon monoxide bound Hb (COHb) instead of oxygen bound Hb (OxyHb); the former being more stable and affords better diffracting crystals, while the latter easily oxidizes to MetHb with poor diffraction (Safo and Abraham, 2001). Note that COHb, OxyHb, and MetHb have same tertiary/quaternary conformations and therefore it is justified to use the more stable COHb for structural studies. Experiments for obtaining DeoxyHb T-state crystals or COHb R-state crystals are described in several published articles (Perutz et al., 1968; Fermi, 1975; Safo et al., 2001; Safo et al., 2004; Pagare et al., 2018; Abdulmalik et al., 2020; Ahmed et al., 2020; Pagare et al., 2020; Alhashimi et al., 2022; Huang et al., 2022). Crystals of Hb normally appear within 2–10 days, and vary in size from microscopic to very large in any direction, and the most common crystal systems observed with Hb are monoclinic, orthorhombic, trigonal and tetragonal (Safo and Abraham, 2001; Safo et al., 2001; Safo et al., 2004; Deshpande et al., 2018; Pagare et al., 2018; Abdulmalik et al., 2020; Pagare et al., 2020; Alhashimi et al., 2022; Huang et al., 2022).

The co-crystallization technique is the most widely used method to obtain co-crystal structures of Hb in complex with allosteric effectors (Safo et al., 2001; Safo et al., 2004; Deshpande et al., 2018; Pagare et al., 2018; Pagare et al., 2020; Alhashimi et al., 2022; Huang et al., 2022). The protein solution and ligand are mixed together and incubated for a certain period of time and then used for the crystallization experiment. It is important to point out that structure-based SCD drug discovery, have in most instances used normal Hb for co-crystallization with the pharmacologic agents. The use of normal Hb instead of sickle Hb is justified as the two structures are similar (Safo et al., 2001; Safo et al., 2004; Ghatge et al., 2016; Metcalf et al., 2017; Abdulmalik et al., 2020; Pagare et al., 2020). DeoxyHb crystals are prepared and mounted under inert atmosphere, usually in the glove box to prevent being oxidized to MetHb (Safo et al., 2001). Following, cryo-loop protected mounted DeoxyHb crystals are flash-frozen by storing in liquid nitrogen until diffraction data collection at cryogenic temperature, which is required to prevent rapid decay but also to keep the protein from oxidizing to metHb during data collection. Liganded Hb crystals in the R-state, on the other hand, can be mounted in the air. The flash-frozen step could be skipped for R-state crystals as long as the mounted crystal is used right away for X-ray data collection.

The tertiary or quaternary structures of Hb within the same conformational state are remarkably similar in all species, making possible an efficient means of molecular replacement to solve the crystal structures of new crystal forms. A number of published Hb structures also crystallize isomorphously with known Hb structures, making it possible to use phases from the known structures for structure determination. X-ray crystallography would not have advanced to where it is today without a parallel revolution in computer technology and crystallographic softwares. When the structure of horse Hb was determined, there were no computer refinement programs. Therefore, the atomic positions were refined visually against the electron density map (Perutz et al., 1960). Earlier computer program refinements of Hb structures were done in real space using Diamond’s method (Fermi, 1975; Ladner et al., 1977; Baldwin and Chothia, 1979). Since these early published Hb structures, and with improvement in data collection, computation and crystallographic softwares, there has been an exponential increase in the number of solved Hb structures, including those from human (with or without allosteric effectors), and non-human, e.g., cat, bovine, fish, pig, bar-headed goose, plants, nematodes, *etc*., (Harutyunyan et al., 1995; Knapp et al., 1999; Mueser et al., 2000; Kidd et al., 2001; Safo and Abraham, 2001; Sundaresan et al., 2021). Several Hb variants have also been determined by X-ray crystallography (Smith et al., 1991; Kavanaugh et al., 1992; Kavanaugh et al., 1998; Vasseur et al., 1992; Pechik et al., 1996; Harrington et al., 1997; Vásquez et al., 1998; Tame and Vallone, 2000; Abdulmalik et al., 2004; Ahmed et al., 2020). It is heartening to know that modernization of X-ray crystallography has made the solution of Hb structures routine, allowing for structure-based drug design of allosteric effectors of Hb with pharmacologic implication.

## 2 Sickle cell disease

### 2.1 Pathophysiology of SCD and treatment options

Sickle cell disease is an inherent genetic disorder characterized by sickle shaped erythrocytes or RBCs. The disease currently affects about 100,000 people in the U.S., mostly of African American origin, and about 20 million individuals worldwide (Chaturvedi and DeBaun, 2016; Piel et al., 2017; Thein et al., 2017; Alrayyes et al., 2018; Safo et al., 2021; Pagare et al., 2022). James Herrick, in 1910, first described elongated RBCs in a patient suffering from a severe anemia, which he later referred to as “sickle shaped” (Herrick, 1910) In 1945, Linus Pauling demonstrated by electrophoresis that SCD originated from an abnormality in the hemoglobin molecule, (Pauling and Itano, 1949; Pauling, 1964), coining the term a “molecular disease” (Rees et al., 2010) John Haldane hypothesized that SCD carriers might reflect a selective advantage in protection against malaria caused by *Plasmodium falciparum*, and proposed what we call today the “malaria hypothesis” of SCD (Lederberg, 1999). Ingram et al., were the first to demonstrate that the mutant sickle Hb (HbS) differs from the normal human adult hemoglobin (HbA) by a single amino acid, (Ingram, 1957; Ingram, 1958), that was identified to be a substitution of βGlu6 of HbA with βVal6, forming HbS. Interestingly, deoxygenated HbS (DeoxyHbS) and not oxygenated HbS (OxyHbS) polymerizes (Ghatge et al., 2016). The polymer formation is initiated by a hydrophobic interaction between the βVal6 residue of one DeoxyHbS molecule and a hydrophobic pocket formed by β_2_Ala70, β_2_Phe85, and β_2_Leu88 of an adjacent DeoxyHbS molecule, resulting in the formation of long, rigid and insoluble 14-stranded fibers, which lead to distortion of RBCs into the characteristic sickled shape (Ferrone, 2004). When RBCs sickle, they become brittle and rigid and do not “squeeze” through the narrow vessels of peripheral capillary beds, resulting in vaso-occlusion (VOC), which impairs microvascular blood flow and a cascade of inter-related secondary adverse events, including but not limited to RBC hemolysis, oxidative stress, decreased vascular nitric oxide (NO) bioavailability, and inflammation (Belcher et al., 2003; Aliyu et al., 2008; De Franceschi, 2009; Akinsheye and Klings, 2010; Safo et al., 2021; Pagare et al., 2022). These downstream events lead to extremely painful VOC, chronic endothelial damage, and progressive end-organ injury and dysfunction that ultimately results in morbidity, poor quality of life, and premature mortality (Belcher et al., 2003; Aliyu et al., 2008; De Franceschi, 2009; Piel et al., 2017).

The hypoxia-driven DeoxyHbS polymerization and RBC sickling is made worse by the inherent high concentration of 2,3-DPG and the concomitant increase in DeoxyHbS concentration in sickle RBCs (Poillon et al., 1986; Poillon and Kim, 1990; Jensen, 2009; Rogers et al., 2013). In addition to the primary pathologic interaction involving βVal6, several key secondary interactions between adjacent DeoxyHbS molecules in the fiber, such as those mediated by the surface-located αF-helix residues αAsn78 or βAsp73, are important for stabilizing the polymer (Bunn, 1986; Eaton and Hofrichter, 1990; Cretegny and Edelstein, 1993; Harrington et al., 1997; Ferrone, 2004; Pagare et al., 2022). Consistently, SCD individuals with a rare second mutation, αAsn78→Lys (Hb Stanleyville) or βAsp73→Val (Hb Mobile), on the αF-helix of Hb show reduced tendency for HbS polymerization and RBC sickling, resulting in mild or even no disease sequelae (Benesch et al., 1979; Rhoda et al., 1983; Burchall and Maxwell, 2010; Pagare et al., 2022). OxyHbS, as noted above neither polymerizes nor incorporates into the polymer fibers since the quaternary conformation does not allow for the pathologic interaction between βVal6 and the hydrophobic acceptor pocket (Ghatge et al., 2016).

Despite the fact that the molecular basis of SCD was established several decades ago, the development of therapeutic agents for SCD has not kept pace. For a very long period, SCD treatment was generally focused on pain management. Hydroxyurea, which induces fetal Hb (HbF) to dilute HbS and thus prevent polymer formation, was the first drug approved in 1998 by the U.S. Food and Drug Administration (FDA) to treat SCD (Telen, 2016; Mvalo et al., 2018; FDA, 2019; Commissioner, 2020). L-glutamine (Endari), with an anti-oxidant effect was approved by the U.S. FDA in 2017 to help neutralize the oxidative stress in sickle RBCs (Niihara et al., 1998; Cieri-Hutcherson et al., 2019; Commissioner, 2020). Crizanlizumab, which was approved in 2019, is a monoclonal antibody targeting P-selectin to reduce erythrocyte adhesion and, consequently, reduce the frequency of VOC crises and complications of SCD (Ataga et al., 2017). Finally, Voxelotor (Figure 2), an aromatic aldehyde developed to prevent the primary pathophysiology of deoxygenation-induced RBC sickling by increasing Hb affinity for oxygen, was approved in 2019 (Metcalf et al., 2017; Dufu et al., 2018; Vichinsky et al., 2019). It is interesting to note that Voxelotor, which is a synthetic analogue of the food flavoring agent, vanillin (Figure 2), was developed based on decades of pioneering work by Abraham and co-workers in the design and development of aromatic aldehydes, including early discovery science with the vanillin derivatives (e.g., INN-312 and SAJ-310; Figure 2) and the furfural, 5-hydroxymethyl-2-furfural (5-HMF; Figure 2), (Abraham et al., 1991; Safo et al., 2004; Abdulmalik et al., 2005; 2011; Safo and Kato, 2014; Oder et al., 2016; Xu et al., 2017; Deshpande et al., 2018; Pagare et al., 2018) underscoring the major and important contribution by Abraham in SCD drug discovery.

### 2.2 Early years of using X-ray crystallography for SCD drug discovery

**FIGURE 4:**
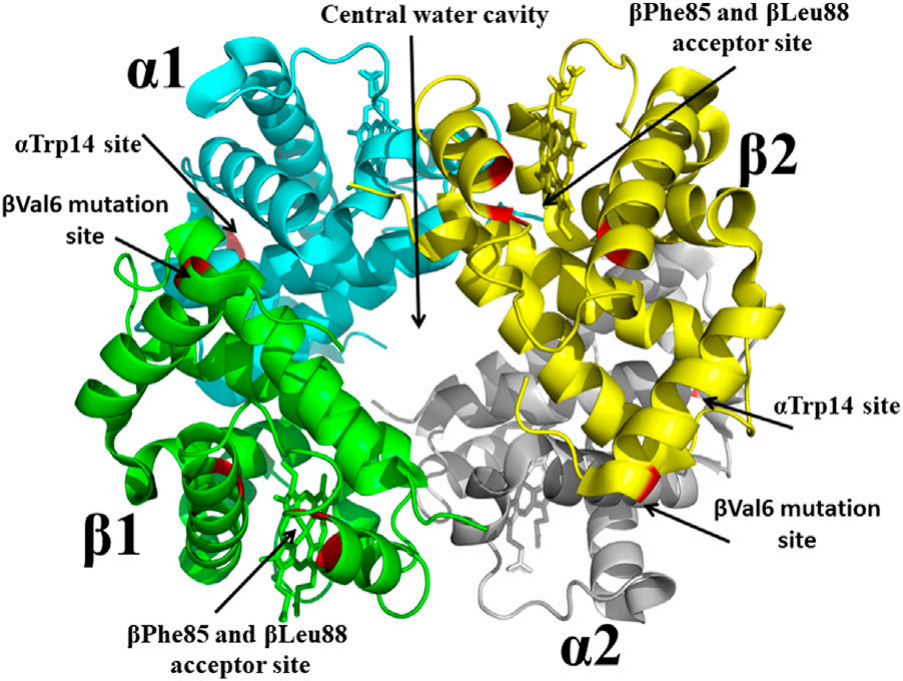
Crystal structure of sickle Hb in the T-state conformation showing targeted binding sites (βVal6 mutation site, βPhe85/βLeu88 acceptor site, central water cavity, and αTrp14 site) for antisickling drug discovery. Hb is shown in ribbon while hemes are shown in sticks.

With the structure determination of liganded and unliganded Hb by Max Perutz in the 1960s and 1970s, (Perutz et al., 1960; Bolton and Perutz, 1970), the dawn of structure-based SCD drug discovery begun, and accelerated by the National Institutes of Health (NIH) request for applications to develop therapeutic agents to treat SCD based on the 3D structures of Hb. It is no wonder that Don Abraham became a pioneer in this burgeoning area of research. In collaboration with Max Perutz and others, Abraham in the 1970s and 1980s using X-ray crystallography and structure-based techniques first focused on finding compounds that target pockets on the surface of Hb, and later compounds that bound to the central water cavity of the protein (Figure 4). Some of these compounds; proline derivatives (e.g., 1-butyryl-4-((carboxymethyl)amino)pyrrolidine-2-carboxylic acid) and substituted alkanoic acid (e.g., 2-((4-bromobenzyl)oxy)acetic acid) (Figure 2), were designed to bind near the βVal6 mutation site or at its receptor cavity in the polymer, βPhe85 and βLeu88 (Figure 4), to directly disrupt the polymer and prevent the βVal6-initiated polymer formation (Abraham et al., 1982; Abraham et al., 1983a; Abraham et al., 1984a; Fatope and Abraham, 1987). None of these compounds showed significant antisickling effect. Don Abraham also designed several antisickling aromatic halogenated acids that were expected to bind to a surface cavity near αTrp14 (Figure 4), and directly inhibit the polymer (Abraham et al., 1984b). One of the studied compounds is Clofibrate (CFA; Figure 2), a marketed antilipidemic agent that was surmised will be ideal candidate as it could be given at high doses daily (2 gm) (Abraham et al., 1983b). Interestingly, and as predicted even though CFA showed an antigelling activity, it decreased the oxygen affinity of hemoglobin, the latter activity, an undesirable property for treating SCD as such low-O_2_ affinity compounds may lead to increase formation of the polymer-forming DeoxyHbS. Low-resolution crystallographic studies suggested three pairs of CFA probably bind to Hb, one at the predicted αTrp14, likely explaining CFA’s antigelling effect (Abraham et al., 1983b). Unexpectedly, two pairs of CFA (4 molecules) bound in the central water cavity of Hb, and located ∼20 Å away from the β-cleft binding site of 2,3-DPG (Abraham et al., 1983b). The binding at the central water cavity of DeoxyHb tied the two heterodimers together, stabilizing the T-state relative to the R-state; explaining CFA’s property of decreasing Hb affinity for oxygen. Due to its ability to decrease the oxygen affinity of Hb, and presumably increase tissue oxygenation, CFA was studied for use by radiation oncologists for the treatment of cancer (Hirst et al., 1987; Hirst and Wood, 1989; Schmeel et al., 2016).

From a drug development perspective, Hb surface binders faced many challenges in becoming effective pharmaceutical products. For example, the surface cavities, e.g., the βVal6 mutation site or the βPhe85 and βLeu88 acceptor site or the αTrp14 site (Figure 4), are too shallow to bind compounds with high affinity, and coupled with the fact that since large amount of drug would be required to modify the large amount of Hb (5 mmol) to reach therapeutic level, these compounds would likely exceed toxicity thresholds. Even though this earlier work by Abraham, targeting surface cavities of Hb, failed to find a promising therapeutic agent, the research laid the foundation for several future novel findings by Abraham and other groups. One such discovery, which continuous to have a scientific impact, is the hemoglobin allosteric effector, RSR-13 (aka Efaproxiral) (Figure 2), which binds to the central water cavity of Hb, (Figure 4), and potently decreases Hb affinity for oxygen (Randad et al., 1991; Abraham et al., 1992; Khandelwal et al., 1993; Phelps Grella et al., 2000; Safo et al., 2001; Safo et al., 2002a; Youssef et al., 2002). Additionally, these earlier work by Abraham set the stage for the discovery of several potent antisickling agents, including Voxelotor.

### 2.3 Discovery of efaproxiral

**FIGURE 5:**
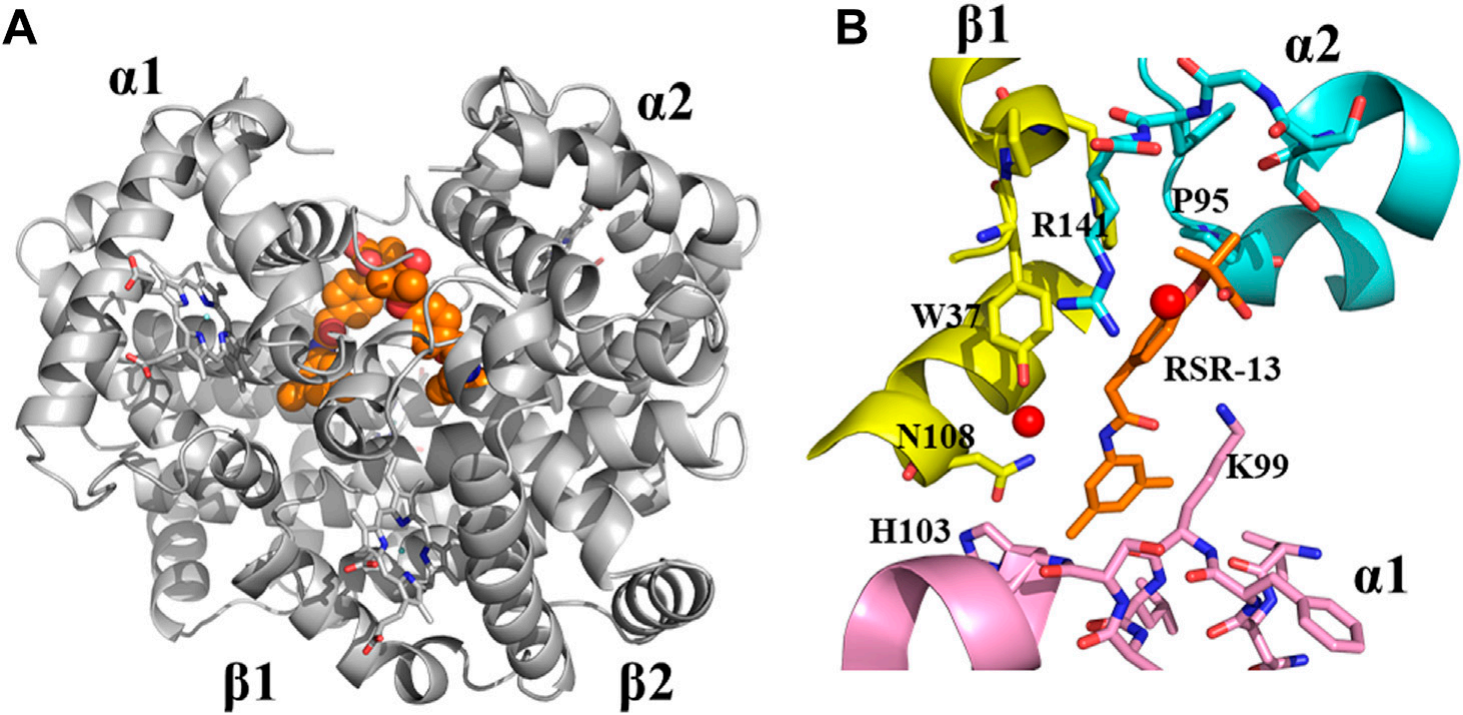
Crystal structure of deoxygenated T-state Hb in complex with two molecules of RSR-13 bound at the central water cavity. **(A)** A global view of RSR-13 molecules (orange spheres) in complex with Hb (gray ribbon). **(B)** A close-up view of RSR-13 (brown sticks) making hydrogen-bond and hydrophobic interactions with the α1-subunit (magenta ribbon and stick), α2-subunit (cyan ribbon and sticks), and β2-subunit (yellow ribbon and sticks). Water molecules are shown as red spheres. The other RSR-13 molecule (not shown) makes symmetry-related interactions with the protein.

Although, this review article is focused on SCD drug discovery, it will be amiss if we do not mention Don Abraham’s pioneering work on Hb allosteric effectors that shift the OEC to the right and decrease Hb affinity for oxygen, particularly with respect to Efaproxiral (Randad et al., 1991; Abraham et al., 1992). With the discovery that CFA binds to the central water cavity of DeoxyHb to stabilize the T-state Hb and pharmacologically increase O_2_ delivery to tissue, (Abraham et al., 1983b), scientists, and most notably Abraham recognized the importance of structure-based drug design of synthetic allosteric effectors that would have high oral bioavailability, easily traverse RBC, bind with high affinity to Hb, and potently increase Hb oxygen delivery to tissues. Physiologically Hb with a bound 2,3-DPG releases 25%–40% of oxygen, (Safo and Bruno, 2011; Ahmed et al., 2020; Alramadhani et al., 2022), and it was expected that more potent right-shifters would lead to an even more increase of oxygen to tissues, and potentially useful for treating hypoxic underlying diseases, such as angina, stroke, trauma, blood storage, and to enhance radiation treatment of hypoxic tumors (Randad et al., 1991; Abraham et al., 1992; Mehta and Khuntia, 2005; Rosenberg and Knox, 2006; Stea et al., 2006; Safo and Bruno, 2011). The discovery of Efaproxiral involved careful iterative targeted modifications starting from earlier studied analogs, e.g., CFA and bezafibrate through both structure activity relationship (SAR) and SBDD using X-ray crystallography (Abraham et al., 1992; Phelps Grella et al., 2000; Safo et al., 2002a; Youssef et al., 2002; Safo and Bruno, 2011). Like bezafibrate, Efaproxiral bound to Hb in a 2:1 ratio, each molecule spanning two CFA binding sites in the central water cavity of Hb to make hydrophobic and hydrogen-bond interactions with several residues from two α-subunits and one β-subunit of the protein in a symmetry-related fashion (Figure 5) (Phelps Grella et al., 2000; Safo et al., 2001; Safo et al., 2002b; Youssef et al., 2002; Safo and Bruno, 2011). In contrast, 2,3-DPG binds only one molecule at the 2-fold dyad of the β-cleft of Hb, making mainly salt-bridge/hydrogen-bond interactions with the β-cleft residues to stabilize the T-state Hb (Arnone, 1972; Arnone, 1972; Gupta et al., 1979; Richard et al., 1993; Safo et al., 2011). Like 2,3-DPG, Efaproxiral and its analogs binding to DeoxyHb led to significant increase in tissue oxygenation, (Abraham et al., 1992; Khandelwal et al., 1993; Phelps Grella et al., 2000; Youssef et al., 2002; Grinberg et al., 2003; Safo and Bruno, 2011), as well as positive hemodynamic effects due to higher concentrations of circulating oxygen (Kunert et al., 1996). Not surprisingly Efaproxiral was reported to be used in the 2011 Cycling Masters National Championship leading to the national champion being disqualified (SoCalCycling.com, 2012).

Efaproxiral formed the basis of a small company, Allos Therapeutics, which was founded by Don Abraham in 1992, and emerged as a candidate for several clinical applications (Khandelwal et al., 1993; Kunert et al., 1996; Grinberg et al., 2003; Peacock and Lesser, 2006; Stea et al., 2006; Suh et al., 2006; Kaal and Vecht, 2007; Scott et al., 2007; Viani et al., 2009). For example, Efaproxiral underwent Phase II study for pulmonary by-pass surgery, as well as a radiation-sensitizing agent associated to radiotherapy of solid tumors. In a multicenter randomized Phase II clinical study in the treatment of brain metastases originating from breast carcinoma, (Peacock and Lesser, 2006; Kaal and Vecht, 2007), Efaproxiral resulted in an extended survival and an improved quality of life (Scott et al., 2007). In a phase III clinical study, “Radiation Enhancing Allosteric Compound for Hypoxic Brain Metastases (REACH)” Efaproxiral was tested in association with oxygen in whole brain radiation therapy (WBRT), (Suh et al., 2006), with the outcome showing survival improvement. A subsequent meta-analysis, however, refuted the efficacy of Efaproxiral in the treatment of brain metastases (Viani et al., 2009). Following the discovery of Efaproxiral, and based on its crystallographic binding to Hb, Abraham designed several follow-up right-shifting analogs, some showing even more potent allosteric activities than Efaproxiral (Abraham et al., 1992; Phelps Grella et al., 2000; Safo et al., 2002a; Safo et al., 2011; Youssef et al., 2002; Safo and Bruno, 2011). However, none of these compounds have generated the same attention as Efaproxiral.

### 2.4 Early years of aromatic aldehyde-based SCD drug discovery

Aromatic aldehydes have been one of the most widely studied class of compounds for the treatment of SCD. It begun in the late 1970s when Irvin Klotz tested several aromatic aldehydes, including vanillin for their antisickling potentials (Zaugg et al., 1977; Zaugg et al., 1980). Klotz showed that these compounds inhibit erythrocyte sickling by forming Schiff-base adduct with Hb, (Zaugg et al., 1977), however, their actual molecular mechanism of action was not revealed. Even though Klotz was the first to identify the potential of aromatic aldehydes as therapeutic agents, it was Abraham and a group from the pharma company Burroughs Wellcome subsequent study of vanillin and its derivatives that catapulted this class of compounds for treating SCD to the limelight, (Beddell et al., 1984; Kneen, 1985; Merrett et al., 1986; Abraham et al., 1991; Safo et al., 2021; Pagare et al., 2022), which eventually led to the approval of the vanillin derivative, Voxelotor for the treatment of SCD in 2019 (Oksenberg et al., 2016; Metcalf et al., 2017; Vichinsky et al., 2019).

While Abraham was designing compounds to bind to the surface cavities of Hb, the research group from Burroughs Wellcome led by Peter Goodford was also actively developing antisickling aromatic aldehydes to treat SCD (Beddell et al., 1984; Kneen, 1985; Merrett et al., 1986). Based on the classical R-state liganded Hb structure, Peter Goodford designed a series of benzaldehyde-carboxylate compounds, which they proposed would crosslink the two α-subunits through a Schiff-base formation between the aldehyde moiety of the benzaldehyde-carboxylate with the αVal1 amine of one Hb α-subunit, and a salt-bridge interaction between the carboxyl group of the benzaldehyde-carboxylate and the αVal1 amine of the opposite α-subunit (Beddell et al., 1984; Merrett et al., 1986). The mode of binding was expected to stabilize the classical R-state and increase the oxygen affinity of sickle Hb, preventing polymer formation (Beddell et al., 1984; Kneen, 1985; Merrett et al., 1986). Two of the most promising compounds to come out of the study were Tucaresol and Valeresol (Figure 2), however the latter failed the phase I clinical study because of poor oral bioavailability (Fitzharris et al., 1985; Keidan et al., 1986). Tucaresol even though showed extremely good pharmacokinetic properties, including oral bioavailability and very long half-life, (Rolan et al., 1993; Rolan et al., 1995; Arya et al., 1996), it was found to cause immune-mediated toxicity during the phase II clinical studies (Rhodes, 2002).

Although, the *in silico* design of the benzaldehyde-carboxylate compounds by Peter Goodford and associates was based on the classical R-state Hb structure, a subsequent crystallographic study by Abraham with DeoxyHb showed that these molecules rather bind to the α-cleft of T-state DeoxyHb instead of the proposed α-cleft of classical R-state liganded Hb to effect their antisickling activities (Wireko and Abraham, 1991). The crystallographic study led Abraham to propose that the antisickling mechanism of aromatic aldehydes was due to the compounds binding to and destabilizing the T-state Hb, increasing the concentration of the non-polymer forming OxyHbS (Wireko and Abraham, 1991). Two decades later, Don Abraham and Martin Safo correctly elucidated the antisickling mechanism of aromatic aldehydes, which as will be discussed later, was primarily due to binding of these compounds to the R2-state Hb (not the classical R-state Hb) to increase the oxygen affinity of Hb (Safo et al., 2004). Even though aromatic aldehydes did not bind as originally designed by Peter Goodford, the discovery of Tucaresol and Valeresol provided the impetus that structure-based drug design could produce viable clinical candidates in a very acceptable time frame.

Abraham suggested three important things that could be learned from Klotz and Goodford work, as well as his earlier SCD drug discovery research. First, among the several classes of compounds studied for their antisickling activities, aromatic aldehydes appear to be unique since not only do they bind to Hb with higher affinity but show significantly more potent pharmacologic activity (Abraham et al., 1991), as clearly demonstrated by Tucaresol (Arya et al., 1996; Pagare et al., 2022). Second, targeting Hb α-cleft with allosteric effectors appear to be the most pharmacologically viable since Hb surface binders show weak antisickling activities. Finally, because of the failure of Tucaresol, aromatic aldehyde that are food based may have better chance of becoming a pharmacologic agent (Zaugg et al., 1977; Abraham et al., 1991; Safo et al., 2004). With these observations in mind, Abraham revisited and further studied the natural flavoring aromatic aldehyde vanillin, (Abraham et al., 1991), which has previously been reported by Klotz as an antisickling agent (Zaugg et al., 1977). Even though Abrahams’s study with vanillin showed proof of concept *in vitro* and early pre-clinical studies for treating SCD, (Abraham et al., 1991), it became clear that like Valeresol, vanillin was also plagued by metabolic instability, which made it orally nonbioavailable (Godfrey et al., 1999; Pagare et al., 2022). The aldehyde moiety of aromatic aldehydes (-CHO) is the most important structural feature for their pharmacologic activity, as the antisickling activity depends on the compounds ability to form a Schiff-base interaction with the N-terminal αVal1 nitrogen of Hb α-subunits (Godfrey et al., 1999; Pagare et al., 2022). However, oxidative metabolism of the functional aldehyde into acid, e.g., by NAD-dependent aldehyde dehydrogenases (ALDH) in the liver and RBC, (Yoshida et al., 1998; Godfrey et al., 1999; Vasiliou et al., 2000), limits the compounds bioavailability, leading to sub-optimal pharmacokinetic/pharmacodynamic (PK/PD) properties (Safo and Kato, 2014; Oder et al., 2016; Pagare et al., 2022). It is notable that introduction of a hydroxyl group on the benzene ring *ortho* to the aldehyde has led to protection of the aldehyde from oxidative metabolism, resulting in significant improvement in both PK and PD properties as observed with Voxelotor and later compounds (Metcalf et al., 2017; Abdulmalik et al., 2020; Pagare et al., 2020; Pagare et al., 2022).

As the vanillin study was ongoing, and the realization that aromatic aldehydes hold hope for SCD therapy, Abraham decided to test several other small molecule aromatic aldehydes, including 5-formylsalicylic (5-FSA; Figure 2) and several of its benzyloxy-formyl benzoic acid analogs for their antisickling potentials (Abraham et al., 1995; Boyiri et al., 1995; Safo et al., 2011). Unexpectedly, the compounds instead of increasing the oxygen affinity of Hb exhibited the opposite effect of decreasing the protein affinity for oxygen. X-ray crystallographic study with DeoxyHb showed the compounds to bind to the α-cleft of Hb and form Schiff-base interaction with the αVal1 amines in a symmetry-related fashion as predicted (Abraham et al., 1995). What is most interesting is that, the carboxylic acid moiety on the benzaldehyde ring (ortho or para to the aldehyde moiety) was disposed to make a strong inter-subunit salt-bridge interaction with the guanidinium group of αArg141 from the opposite α-subunit, leading to stabilization of the T-state Hb, and explaining the compounds biological activity of decreasing Hb affinity for oxygen. Obviously, these molecules are not candidates for SCD therapy since they will lead to increase formation of the polymer forming DeoxyHbS. Nonetheless, the study gave insight into how aromatic aldehydes bind to the same α-cleft, forming the same Schiff-base interaction with αVal1 amine but exhibit opposite allosteric activities. This discovery started a new program of structure-based bis-aldehyde crosslinkers utilizing X-ray crystallography and structure-based drug design by Abraham to discover several agents, e.g., 5-((2-carboxy-4-formylphenoxy) methoxy)-2-formylbenzoic acid (Figure 2) that cross-link αVal1 amine from one Hb α-subunit and the opposite αLys99 (amine)/αArg141 (guanidinium) to potently decrease Hb affinity for oxygen with potential use as blood substitutes and treatment of hypoxic diseases (Abraham et al., 1995; Boyiri et al., 1995; Safo et al., 2011). Unfortunately, these compounds were not cell permeable.

### 2.5 Development of modern aromatic aldehydes for the treatment of SCD

The late 1990s and early 2000s could be described as the “dark days” of targeting Hb for drug discovery to treat SCD. The 80s had seen the failure of Hb surface binders as antisickling agents. Moreover, several much-touted antisickling aromatic aldehydes, e.g., Valeresol, Tucaresol, and vanillin despite their early promising results had all failed to become therapeutic agents. There was a general concern in the scientific community about the druggability of Hb, primarily due to efficacy and/or toxicity. The mechanism of antisickling action of increasing Hb O_2_-affinity by aromatic aldehydes was also called into question because of the potential for treated cells (high-affinity cells) to “steal” oxygen from untreated cells (low-affinity cells), possibly resulting in adverse effects. Despite these concerns, Abraham and associates continued their quest to target Hb for SCD treatment. Starting with the non-toxic vanillin and application of X-ray crystallography, Abraham and Safo, and their colleagues from the Children’s Hospital of Philadelphia (Toshio Asakura and Osheiza Abdulmalik) made several targeted and iterative modifications where the benzaldehyde moiety was substituted with methoxypyridine, as well as methoxy or alkyl groups (Nnamani et al., 2008; Abdulmalik et al., 2011). Some of the compounds showed as much as 50- to 100-fold potency over vanillin in increasing Hb affinity for oxygen and/or preventing hypoxia-induced RBC sickling (Nnamani et al., 2008; Abdulmalik et al., 2011). Two of the most potent compounds were INN-312 and SAJ-310, (Abdulmalik et al., 2011; Pagare et al., 2018), which interestingly, in addition to their ability to prevent RBC sickling by increasing Hb affinity for oxygen, also appear to directly destabilize the polymer to prevent RBC sickling; an antisickling mechanism that is independent of oxygen. Crystallographic studies showed INN-312 or SAJ-310 and similar analogs to make additional interactions to the protein compared to vanillin, explaining their potent allosteric and antisickling activities (Abdulmalik et al., 2011; Pagare et al., 2018). Importantly, the binding of INN-312 or SAJ-310 directed the pyridine-methoxy moiety (which is *ortho* to the aldehyde group) toward the mouth of the α-cleft to make a weak hydrophobic interaction with the surface-located αF-helix (Abdulmalik et al., 2011; Pagare et al., 2018). The αF-helix has been implicated in stabilizing the HbS fiber by mediating hydrogen-bond interactions between adjacent HbS molecules in the polymer, (Benesch et al., 1979; Rhoda et al., 1983; Burchall and Maxwell, 2010). The interaction between αF-helix and INN-312 or SAJ-310 was proposed to perturb the αF-helix-mediated polymer interaction, explaining the compounds’ O_2_-independent antisickling effect (Abdulmalik et al., 2011; Pagare et al., 2022). Unfortunately, even though these compounds showed significantly improved antisickling activities, they suffered the same fate as Valeresol or vanillin for being orally nonbioavailable because of metabolism of the aldehyde moiety *in vivo* (Fitzharris et al., 1985; Keidan et al., 1986; Oder et al., 2016).

While the rational modification of vanillin was ongoing, Abraham and his colleagues also studied another aromatic aldehyde, 5-HMF and several of its analogs (Safo et al., 2004; Abdulmalik et al., 2005; Kato et al., 2013; Safo and Kato, 2014; Xu et al., 2017; Alhashimi et al., 2022; Pagare et al., 2022). 5-HMF is a by-product of the Maillard reaction that occurs during thermal decomposition of sugars (Hodge, 1953; Hodge, 1955; van Putten et al., 2013; Liu et al., 2018; Lucas et al., 2019). It is present naturally in many foods and drinks such as coffee, caramel, fruits and honey. 5-HMF showed remarkable *in vitro* antisickling biological effects (Safo, 2004; Safo et al., 2004; Abdulmalik et al., 2005; Kato et al., 2013; Safo and Kato, 2014; Kato, 2016). Like vanillin and other aromatic aldehydes, 5HMF forms Schiff base-adduct with Hb by interacting with the two α-chain αVal1 amines that led to increase in Hb affinity for oxygen; preventing hypoxia-induced RBC sickling. 5-HMF also improved the survival of transgenic (Tg) “sickle” mice after a hypoxic challenge (Abdulmalik et al., 2005). It was during the study of 5-HMF that aromatic aldehydes’ true antisickling mechanism of action was elucidated, which revolutionized and speeded development of aromatic aldehyde structure-based SCD drugs (Safo et al., 2004). As noted above, Peter Goodford using *in silico* study has assumed that aromatic aldehydes bind to liganded Hb in the classical R-state conformation to increase Hb affinity for oxygen, (Beddell et al., 1984), while Abraham through crystallographic study suggested that these compounds biological effect is due to binding to the DeoxyHb and destabilizing the T-state to increase Hb affinity for oxygen (Wireko and Abraham, 1991). However during the 5-HMF study, it was realized that aromatic aldehydes primarily bind to the R2-state Hb structure and not the classical R-state Hb, the latter α-cleft too small to fit the compounds, to stabilize the relaxed state Hb and increase Hb affinity for oxygen (Safo et al., 2004; Abdulmalik et al., 2005; 2011). The compounds also even though bind to DeoxyHb as previously suggested by Abraham, the binding is significantly weaker, and probably does not contribute significantly to the allosteric potential of the compounds (Safo et al., 2004; Abdulmalik et al., 2005; Abdulmalik et al., 2011).

5-HMF progressed through phase I/II clinical trials in healthy volunteers and adults with SCD under the NIH Therapeutics for Rare and Neglected Diseases Program, TRND (ClinicalTrials.gov Identifier NCT01597401) (Stern et al., 2012; Safo et al., 2021; Pagare et al., 2022) The study conducted by Dr. Gregory Kato at the NIH/NHLBI and AesRx LLC showed significant improvement in several clinical outcomes, including reduced pain (with synergistic effect when used with HU), decreased RBC hemolysis, reduction in blood pressure, and increase in blood oxygen levels (S_p_O_2_) during hypoxia challenge (ClinicalTrials.gov identifier NCT01597401) (Stern et al., 2012; Mendelsohn et al., 2013; Oder et al., 2016; SAIC-Frederick, Inc., 2021) Even though Abraham had retired from active research during the latter part of 5-HMF clinical study, it is an understatement that he was very elated with the phase I/II clinic result, which is captured in one of his interviews, and I quote, “I threw everything, my whole heart and science into sickle cell disease. For a while it was not the wisest thing to do*.* But I so wanted to use structural biology to develop a drug. I started to discover a drug to treat sickle cell disease in 1975. Since that time, my research groups at the University of Pittsburgh and Virginia Commonwealth University have spent nearly 40 years in pursuit of an agent to treat sickle cell anemia. We have also collaborated with numerous researchers at US universities, and internationally at and with some of the most renowned laboratories and scientists, i.e., the Medical Research Council Laboratory of Molecular Biology (MRCLMB) Cambridge United Kingdom, INSERM in Paris, and the University of Parma in Italy. It is fair to say that our research group has conducted the longest ongoing research in the world to attempt to discover an effective anti-sickling agent. My firm conviction from my many years of studying sickle cell anemia is that 5-HMF provides the highest chance of clinical success of any agent yet discovered.”

Don Abraham retired from VCU in 2007, as the phase I/II clinical study with 5-HMF begun to wind down. Unfortunately, the phase II study did not fare well and was terminated due to several reasons: First, due to low oral bioavailability as a result of extensive oxidative metabolism of 5-HMF in humans; second due to premature unblinding of the clinical data results, and lastly due to the reported study of Voxelotor, which showed this compound to be significantly more potent, and most importantly significantly better oral bioavailability than 5-HMF (Kato, 2016; Metcalf et al., 2017; Dufu et al., 2018; Vichinsky et al., 2019; Safo et al., 2021; Pagare et al., 2022). As noted above, Voxelotor would go on to be approved for the treatment of SCD (Vichinsky et al., 2019). Although 5-HMF failed in the clinical study, the findings, coupled with those from the vanillin derivatives and Efaproxiral studies once again provided a strong rationale and validity for the concept of aromatic aldehydes for SCD therapy, as well as justification for a structure-based approach to design potent aromatic aldehydes that exert their therapeutic effects via multiple mechanisms of action and improved PK profiles. For example, the outcomes of Don Abraham’s study spurred further investigations into improving the metabolic stability of aromatic aldehydes, as well as discovering antisickling mechanism(s) beyond increasing Hb affinity for oxygen.

### 2.6 Targeted identification of dual acting antisickling agents for sickle cell disease therapy

Although SCD is caused by a single-point mutation, multiple downstream pathways are affected, with each contributing to the pathogenesis of the disease (Kato et al., 2007; Rees et al., 2010; Chaturvedi and DeBaun, 2016; Habara and Steinberg, 2016; Thein et al., 2017; Ware et al., 2017; Alrayyes et al., 2018; Safo et al., 2021; Pagare et al., 2022). This complexity poses a fundamental challenge for therapeutics focused on mitigating a single pathologic process. There are two key mechanisms by which the tendency for polymerization of HbS may be reduced: (1) decreasing the intracellular concentration and/or fraction of polymer-forming HbS by increasing the oxygen affinity of HbS (O_2_-dependent antisickling mechanism), or (2) directly destabilizing polymer formation (O_2_-independent antisickling mechanism) (Safo et al., 2021; Pagare et al., 2022). While Voxelotor approval sets the stage for broad adoption of the O_2_-dependent therapeutic modality, (Oksenberg et al., 2016; Metcalf et al., 2017; Dufu et al., 2018; Safo et al., 2021; Pagare et al., 2022), this antisickling mechanism has limitations. The allosteric effect of increasing O_2_ affinity is inherently limited by the need to avoid impeding O_2_ unloading to tissues. Moreover, Voxelotor bound HbS tetramers even though may be incorporated into fibers in areas of severe regional hypoxia may not exhibit any pharmacologic effect since it required presence of oxygen for its pharmacologic activity.

**FIGURE 6:**
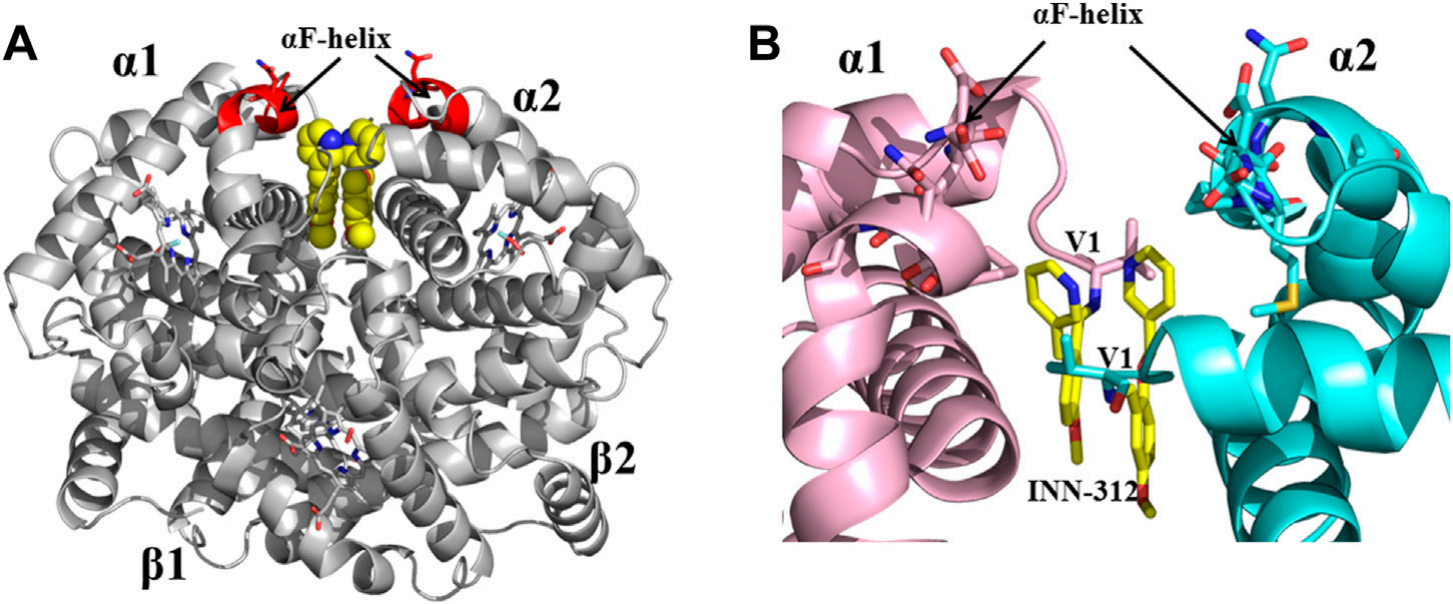
Crystal structure of liganded Hb (in the R2-state conformation) in complex with two molecules of INN-312 bound at the α-cleft. **(A)** A global view of INN-312 molecules (yellow spheres) in complex with Hb (gray ribbon). The surface-located αF-helix is shown in red ribbon, and hemes shown in sticks. **(B)** A close-up view of Schiff-base interaction between INN-312 (yellow sticks) and Hb αVal1 amine, as well as close interactions with the αF-helix residues (sticks). Other protein interactions are not shown for clarity. α1-subunit and α2-subunit are shown in pink and cyan, respectively.

**FIGURE 7:**
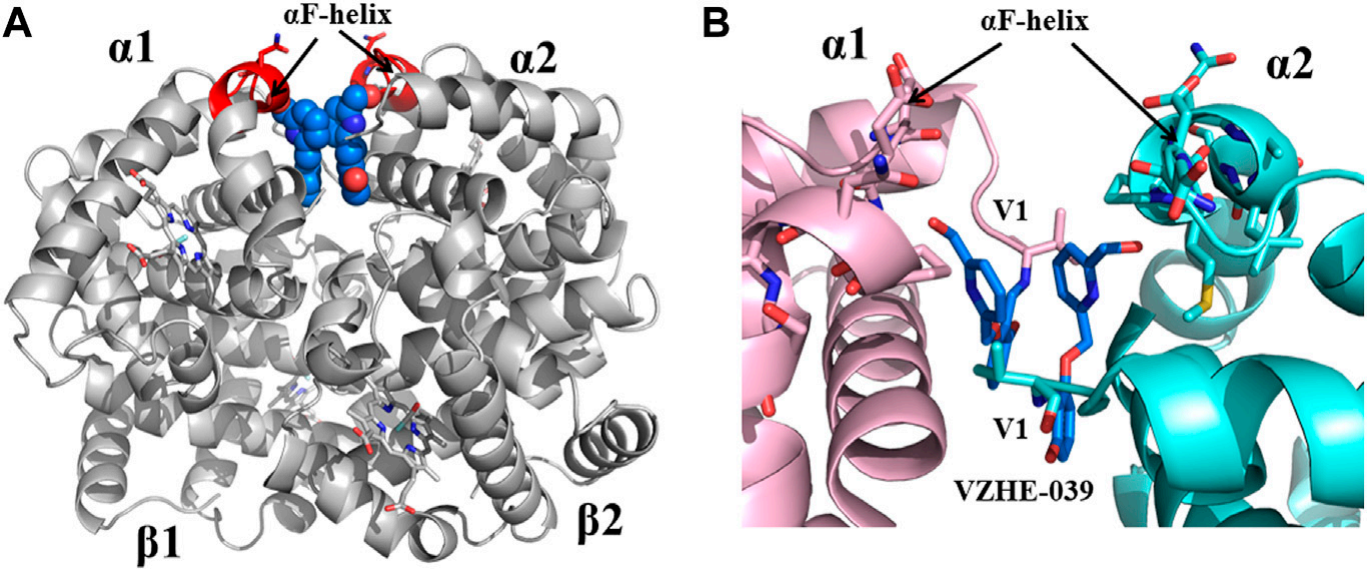
Crystal structure of liganded Hb (in the R2-state conformation) in complex with two molecules of VZHE-039 bound at the α-cleft. **(A)** A global view of VZHE-039 molecules (marine blue spheres) in complex with Hb (gray ribbon). The surface-located αF-helix is shown in red ribbon, and hemes shown in sticks. **(B)** A close-up view of Schiff-base interaction between VZHE-039 (marine blue sticks) and Hb αVal1 amine, as well as close interactions with the αF-helix residues (sticks). Other protein interactions are not shown for clarity. α1-subunit and α2-subunit are shown in pink and cyan, respectively.

Previous studies by Abraham and Safo have identified several novel compounds, e.g., INN-312, which not only prevented RBC sickling through O_2_-dependent antisickling mechanism of action as Voxelotor, but also showed weak direct polymer destabilization effect, a unique O_2_-independent antisickling property (Abdulmalik et al., 2011). With this knowledge in mind, Safo (mentored by Abraham) and his collaborative team begun a systematic structure-based drug discovery to develop next generational compounds that when bound to HbS tetramers (like fetal Hb tetramers), would resist co-polymerization not only due to increased O_2_ affinity, but also because of destabilization of HbS intermolecular contacts that are critical to the stability of insoluble fibers (Abdulmalik et al., 2011; Deshpande et al., 2018; Pagare et al., 2018; Abdulmalik et al., 2020; Pagare et al., 2020; Safo et al., 2021; Pagare et al., 2022). Starting with the crystallographic binding mode of INN-312 at the α-cleft of Hb that showed the ortho-positioned methoxy-pyridine substituent making weak hydrophobic interactions with the surface-located Hb αF-helix (Figure 6), the group work spanning several years discovered very potent dual antisickling compounds, with examples as PP-14 and VZHE-039 (Figure 2) (Abdulmalik et al., 2011; Abdulmalik et al., 2020; Pagare et al., 2020; Pagare et al., 2022) PP-14 and VZHE-039 bind to the α-cleft of R2-state Hb in a similar fashion as INN-312, however, these two compounds made closer and stronger hydrogen-bond interactions with the αF-helix compared to INN-312 (Figure 7), resulting in potent antisickling effect even in the absence of oxygen when compared to INN-312. Some of these compounds are undergoing IND-enabling studies for the treatment of SCD. As noted above, the αF-helix is known to be an important polymer stabilizer, (Benesch et al., 1979; Rhoda et al., 1983; Bunn, 1986; Burchall and Maxwell, 2010), and thus weakening the αF-helix mediated polymer stabilization interactions is expected to lead to antisickling effect (Pagare et al., 2020; Pagare et al., 2022; Safo et al., 2021). The theoretical basis for this novel antisickling mechanism comes from individuals of Sudanese and Congolese ancestry who inherited a rare double mutant Hb variant, referred to as HbS Stanleyville II, possessing both the classic HbS (βGlu6→βVal6) mutation and an amino acid substitution (αAsn78→αLys78) on the surface of the Hb αF-Helix (Rhoda et al., 1983; Burchall and Maxwell, 2010). Like Hb Stanleyville individuals with benign disease, it is expected that these dual antisickling compounds with several innovative features would offer a unique and promising approach to SCD treatment that is superior to existing options to combat SCD pathophysiology.

## 3 Conclusion and perspective

Here we present how X-ray crystallography and SBDD have played a pivotal role in the discovery of antisickling agents for the treatment of sickle cell disease, and to improving the pharmacologic properties of next-generation antisickling drug candidates. We also present the major and important role played by Donald Abraham in this field. It all started in the late 70s, when Don Abraham had a vision to use X-ray crystallography and crystal structures of hemoglobin for structure-based drug discovery to develop antisickling drugs to treat SCD. He was on a quest to find a drug to treat the primary pathophysiology of the disease even though he knew it was not going to be easy. But against-all-odds and obstacles, false starts, dogged determination, he made significant contribution in this field. Abraham’s groundbreaking work in SCD drug discovery provided the foundation for the SBDD paradigm, which has successfully been used not only to develop antisickling agents, but also to develop pharmacologic agents for a myriad of diseases. He took three Hb-targeting drugs to the clinic, two for the treatment of SCD that include vanillin and 5-HMF, and a third Efaproxiral for the treatment of hypoxia-underlying diseases. The work by Abraham has also led to many good lessons being learned; his early findings with structure-based drug design have enabled better design of next generational compounds for SCD, and credit should be given to him for the eventual discovery of Voxelotor, which was based on one of his earlier discovery, INN-312. Finally, the impact of his mentoring of so many young scientists, e.g., Safo and Abdulmalik had been enormous, by spurring these generational scientists on a quest to discover superior drugs to treat SCD.
